# Supplementary figures and images for: Integrated Design and Fabrication of Pneumatic Soft Robot Actuators in a Single Casting Step
Source: Cyborg Bionic Syst. 2024 Jul 17;5:0137. doi: 10.34133/cbsystems.0137 (PMC11254383; doi:10.34133/cbsystems.0137)

**A**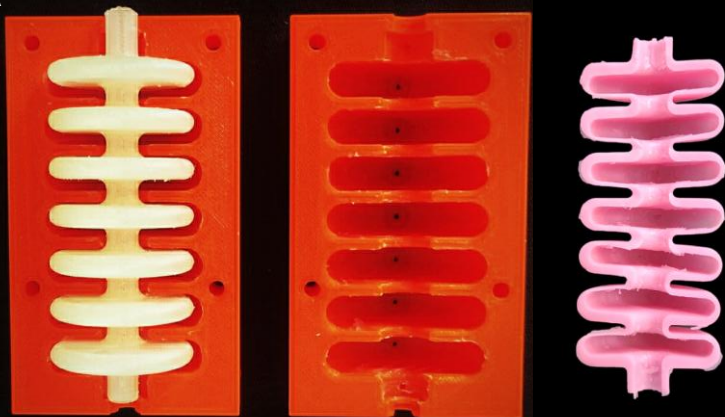**B**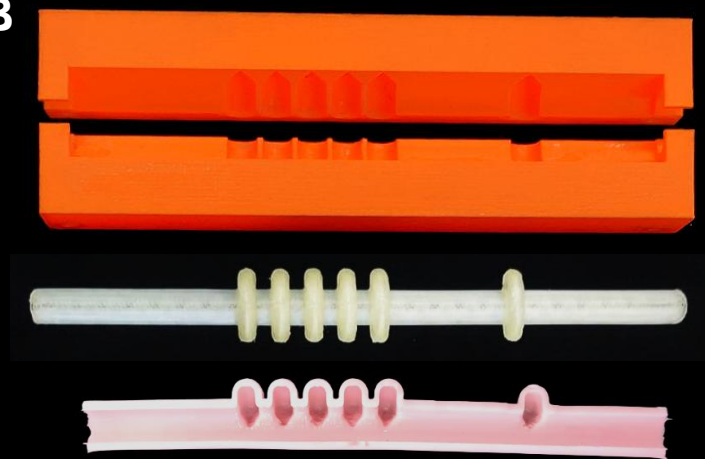**C**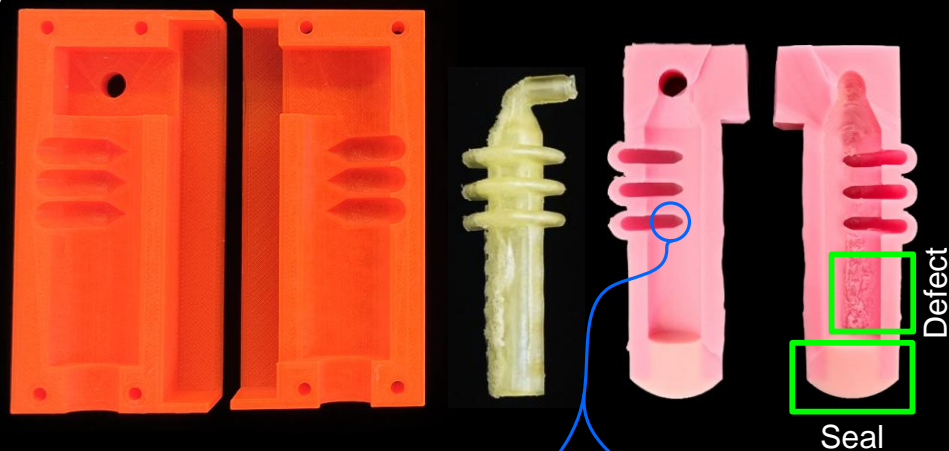**D**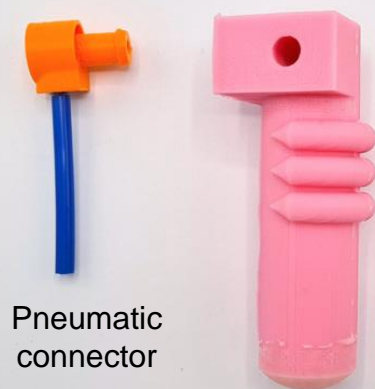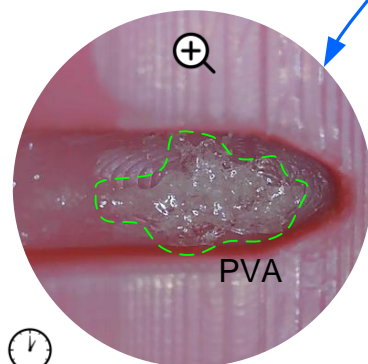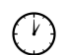

5 min of heated water flow

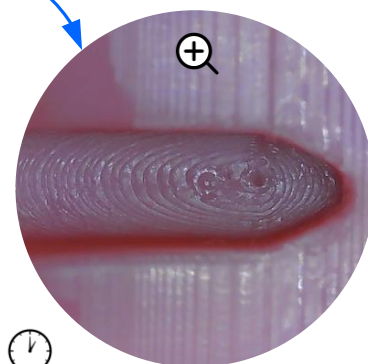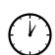

20 min of heated water flow

Supplement: Supplementary 1 — Figs. S1 and S2 Movies S1 to S5 [file cbsystems.0137.f1.zip › figS1.pdf]

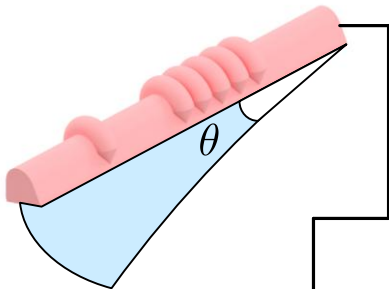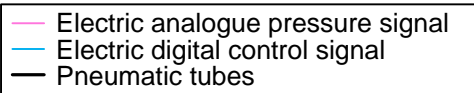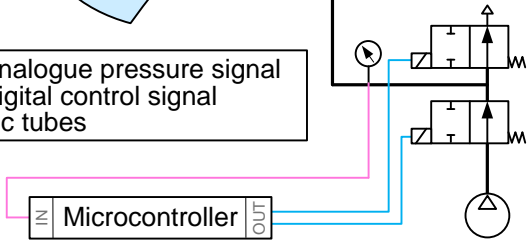

Supplement: Supplementary 1 — Figs. S1 and S2 Movies S1 to S5 [file cbsystems.0137.f1.zip › figS2.pdf]
